# Uncertainty Breeds Anxiety and Depression: The Impact of the Russian Invasion in Ukraine on a Swedish Clinical Population Receiving Internet-Based Psychotherapy

**DOI:** 10.32872/cpe.12083

**Published:** 2024-03-28

**Authors:** Jón Ingi Hlynsson, Oskar Gustafsson, Per Carlbring

**Affiliations:** 1 Department of Psychology, Stockholm University, Stockholm, Sweden; 2 Department of Statistics, Stockholm University, Stockholm, Sweden; Philipps-University of Marburg, Marburg, Germany

**Keywords:** anxiety, depression, Russian–Ukrainian war, uncertainty-inducing event, clinical trial, internet-based psychotherapy, emotional disorders

## Abstract

**Background:**

Recent global crises, such as the COVID-19 pandemic and the 2022 Russian invasion of Ukraine, have contributed to a rise in the global prevalence of anxiety and depressive disorders. This study examines the indirect impact of the Ukraine war on emotional disorders within a Swedish clinical population.

**Method:**

The sample comprised participants (n = 1,222) actively engaged in an internet-based psychotherapeutic intervention (cognitive-behavioral, psychodynamic, and waitlist) when the war broke out. The Patient Health Questionnaire-9 scale and the Generalized Anxiety Disorder-7 scale were used to measure depression and anxiety.

**Results:**

Anxiety and depressive symptom severity increased following the war's onset, with an average weekly increase of 0.77-points for anxiety (p = .001, Cohen's d = 0.08) and 0.09-points for depression (p = .70, Cohen's d = 0.01); however, the increase was negligible for depression. Furthermore, higher socioeconomic status (SES) predicted declines in depression and anxiety during the study period, with a 0.69-point average weekly decrease in anxiety (p < .001, Cohen's d = 0.32) and a 1.09-point decrease in depression (p < .001, Cohen's d = 0.48) per one unit increase in SES, suggesting that SES may serve as a protective factor that buffers against psychopathological development during crises.

**Conclusions:**

These findings have implications for mitigating the development of psychopathology during crises and interpreting treatment efficacy estimates during such events. Our findings also emphasize the potential of internet-based psychotherapy in addressing emotional disorders during crises. This study presents up-to-date information about the reaction of treatment-seeking individuals to abrupt uncertainty.

## Highlights

- The 2022 Russian invasion of Ukraine rapidly exacerbated anxiety symptom severity.
- Socioeconomic status may buffer against psychopathology during heightened uncertainty.
- Spatially distant uncertainty-inducing events can elevate the risk for psychopathology.
- Increased anxiety during crises may confound treatment efficacy estimations.

In recent years, the world has faced numerous global crises with devastating consequences for mental health. For instance, depression prevalence rose significantly after the 2008 global financial crisis (Guerra & Eboreime, 2021), and anxiety and depression rates worldwide increased by roughly 25% during the COVID-19 pandemic (Ettman et al., 2020; World Health Organization, 2022). Similarly, the Russian invasion of Ukraine on February 24th, 2022, resulted in increased prevalence rates of anxiety and depression among Ukrainians (Osokina et al., 2023; Xu et al., 2023) and Europeans (Riad et al., 2022; Skwirczyńska et al., 2022). Although these crises differ, they share a common characteristic: an increase in symptomatology of emotional disorders in response to an increase in external uncertainty.

Emotional disorders are characterized by frequent experiences of negative emotions, along with maladaptive reactions to and regulation of these experiences. These maladaptive reactions contribute to the persistence of negative emotions and the maintenance of the presenting disorder symptoms (cf. negative feedback loop; Bullis et al., 2019). Effectively managing uncertainty is already a critical adaptive challenge for humans. However, when environmental uncertainty abruptly increases, as during a global pandemic or war outbreak, adaptive information processing becomes even more hindered by internal disorder and uncertainty. These features, known as psychological entropy (Hirsh et al., 2012), tend to increase during crises, in turn, raising the likelihood of psychopathological development. For instance, anxiety and depressive symptoms were significantly higher during the COVID-19 pandemic compared to pre-pandemic rates (Gao et al., 2020; Xiong et al., 2020), with worldwide prevalence rates rising by 25% (Ettman et al., 2020; World Health Organization, 2022) and pandemic-related media exposure increased the odds of presenting with anxiety and combined anxiety and depression (Gao et al., 2020). Similarly, economic recessions (e.g., the 2008 global financial crisis) are associated with an overall increase in depression and anxiety prevalence rates, with low socioeconomic status as a significant risk factor (Frasquilho et al., 2016; Gili et al., 2013; Guerra & Eboreime, 2021).

Focusing on the recent^1^1It should be noted that tensions between Russia and Ukraine began in 2014, but escalated into a full-blown war in February 2022, following Russia's invasion of Ukraine (cf. Michailova, 2022). war outbreak in Ukraine, a study by Riad et al. (2022) found that Czech university students reported high levels of concern about the ongoing conflict, with increased age correlating with higher levels of concern and media exposure engagement predicting anxiety and depression severity. Similarly, Skwirczyńska et al. (2022) discovered a positive association between war-related fear and anxiety severity in a Polish student sample. Intriguingly, access to monetary savings emerged as a protective factor that reduced the odds of presenting anxiety symptoms. One interpretation of Skwirczyńska et al.'s (2022) findings is that socioeconomic status, as indicated by access to monetary savings, buffers against anxiety symptom development (cf. Guerra & Eboreime, 2021). In summary, the war outbreak in Ukraine has noticeably affected the European population.

## Internet-Based Therapy

In recent years, a disparity has emerged between the demand for psychotherapy and its availability. As a result, the utilization of internet-based psychotherapeutic interventions has risen substantially to address this gap (Andersson et al., 2019). Internet-based psychotherapeutic treatments leverage technological advancements to create a contemporary alternative to traditional therapy. Typically, internet-based therapy consists of structured, manualized psychotherapy delivered online through modules containing self-help texts and the option to communicate with a therapist via encrypted messages (Andersson & Carlbring, 2022). Designed to parallel conventional face-to-face therapy in length and content (Andersson et al., 2016), internet-based therapy demonstrates equivalent overall therapeutic efficacy (Hedman-Lagerlöf et al., 2023). Meta-analytic findings support the treatment efficacy of internet-based therapy for emotional disorders, revealing moderate to large effect sizes for anxiety and depressive disorders (Andersson et al., 2019; Hedman-Lagerlöf et al., 2023).

## Aim of the Present Study

This study aims to assess the effects of indirect experiences of the war outbreak in Ukraine on the severity of anxiety and depression among individuals seeking treatment through an internet-based intervention, hereafter collectively referred to as "treatment-seeking individuals". Although this study was conducted in Sweden, which is a neighboring country but not directly bordering Ukraine (i.e., approximately 1500 kilometers separate Sweden and Ukraine), previous studies suggest that the war outbreak in Ukraine has increased the prevalence rates of anxiety and depression in the general European population (Riad et al., 2022; Skwirczyńska et al., 2022). Indeed, surges in exposures to psychological threats (e.g., media exposure to crisis-related content) can jeopardize individuals' sense of personal security and exacerbate psychopathological development (Gao et al., 2020; Jayuphan et al., 2020; Riad et al., 2022). Consequently, we predicted a divergence in weekly therapeutic efficacy trends among treatment-seeking individuals following the war outbreak, as indicated by a spike in anxiety and depression. To our knowledge, this is the first study investigating the effects of the war in Ukraine on emotional disorders in a clinical population and thereby aims to provide up-to-date information about the reaction of treatment-seeking individuals to abrupt uncertainty.

## Hypotheses

This study has two core hypotheses: Scores on the 1) PHQ-9 and 2) GAD-7 will be significantly elevated following the outbreak of war in Ukraine when compared to a baseline established by the trend in scores observed over the preceding four weeks, adjusting for treatment group assignment, socioeconomic status, education level, age, and gender. Additionally, high socioeconomic status is hypothesized to be a protective factor that buffers against further development of psychopathology following the war outbreak.

## Method

### Participants and Recruitment

The present study utilizes data from an ongoing clinical trial (ClinicalTrials.gov identifier: NCT05016843) that is being conducted in Sweden. Participants were recruited online through a website outlining the study's aims and components (Vlaescu et al., 2016). The study was advertised on Facebook and also spread through word of mouth. Participants did not receive any monetary compensation for their involvement in the study. The only form of compensation provided was the inherent benefits derived from participation in the treatment interventions. See Figure S1, Supplementary Materials, for a flow chart illustration of the study design.

#### Sample Size

All participants (*n* = 1,222) actively engaged in the study between January 24th, 2022, and March 24th, 2022, were included. This two-month period was chosen to adequately represent treatment efficacy before and after the war outbreak in Ukraine on February 24th, 2022.

#### Eligibility Criteria

Eligibility criteria were assessed during the study's screening phase. Participants were required to: a) be at least 18 years of age; b) read and write in Swedish; c) have an internet connection via their mobile phone or computer; and d) experience at least mild anxiety symptoms (i.e., GAD-7 ≥ 5 points) or mild to moderate depression symptoms (i.e., PHQ-9 ≥ 10 points), or both. Participants were excluded if they: a) were currently seeking other psychological treatment; b) had begun or adjusted psychopharmacological treatment for anxiety, worry, or depression within the nearest month from screening; or c) had severe depression (i.e., PHQ-9 ≥ 20 points) or suicidality (i.e., PHQ-9, item nine score > 2 points) indicated during screening.

### Measures

Demographic variables and anxiety and depression measurements were collected during screening, followed by weekly measurements of anxiety and depression.

#### Demographics

Demographic variables collected during screening included age, gender, socioeconomic status^2^2Socioeconomic status was indirectly measured with a self-rated scale; participants rated their socioeconomic status in relation to others on a scale from 1 to 5 (see Table 1 for response options)., marital status, household composition, level of education, employment status, mental health characteristics, and prior psychopharmaceutical medication usage.

#### Patient Health Questionnaire 9-Item Scale (PHQ-9)

The Patient Health Questionnaire 9-item scale (PHQ-9) is a self-report questionnaire that quantifies depression severity (Kroenke et al., 2001). Each item is rated on a scale from 0 to 3, with total scores ranging from 0 to 27. A score of 10 or higher is a diagnostic indicator of depression (Kroenke et al., 2001, 2010). The PHQ-9 consistently demonstrates good accuracy and discrimination ability in clinical settings and the general population (Kocalevent et al., 2013; Kroenke et al., 2001, 2010) as well as when administered via the internet (Martin-Key et al., 2022). In this study, the PHQ-9 exhibited adequate internal reliability during screening, Cronbach's alpha = 0.66, 95% CI [0.63, 0.68], indicating acceptable internal consistency. It should be noted that this internal consistency reliability estimate suffers from a restriction of range and an analysis of the whole sample at screening (both included and excluded participants) yielded Cronbach's alpha = 0.81, 95% CI [0.80, 0.62] (Hlynsson & Carlbring, 2023).

#### Generalized Anxiety Disorder 7-Item Scale (GAD-7)

The Generalized Anxiety Disorder 7-item scale (GAD-7) is a self-report questionnaire that assesses anxiety and screens for generalized anxiety disorder (Spitzer et al., 2006). Each item is rated on a scale from 0 to 3, with total scores ranging from 0 to 21. A score of 8 or higher is a diagnostic indicator of anxiety disorders (Luo et al., 2019; Spitzer et al., 2006). The items align with DSM-5 criteria (American Psychiatric Association, 2022) and are sensitive to various anxiety disorders (Kroenke et al., 2010) in both clinical settings and the general population, as well as when administered online (Byrd-Bredbenner et al., 2021; Johnson et al., 2019; Löwe et al., 2008; Martin-Key et al., 2022). In this study, the GAD-7 demonstrated good internal reliability during screening, Cronbach's alpha = 0.77, 95% CI [0.75, 0.79], indicating excellent internal consistency. It should be noted that this internal consistency reliability estimate suffers from a restriction of range and an analysis of the whole sample at screening (both included and excluded participants) yielded Cronbach's alpha = 0.85, 95% CI [0.83, 0.85] (Hlynsson & Carlbring, 2023).

### Treatment Interventions

Data was collected as part of an ongoing clinical trial (Mechler et al., 2022) comparing cognitive-behavioral therapy (unified protocol [UP]; Barlow et al., 2017) with psychodynamic affective phobia (AP) therapy (Julien & O’Connor, 2017). The trial comprised three factors: a) type of internet-based treatment intervention; b) treatment length; and c) effects of access to a clinician-moderated discussion forum. Participants were randomly assigned via a factorial assignment mechanism to one of twelve conditions: UP, AP, or a waitlist, each for either 8 or 16 weeks, and each with or without access to a clinician-moderated forum.

### Data Analysis

The data was analyzed using R Studio (R Core Team, 2021). A panel-data regression analysis was conducted, in which PHQ-9 and GAD-7 scores were separately predicted by the treatment time course in weeks (e.g., data provided between January 24th and January 30th, 2022, was assigned the number 1 corresponding to week one) and a dummy variable containing information about whether data corresponded to the time period before or after the war outbreak (i.e., all data corresponding to dates before February 24th, 2022, was coded as 0 and other data as 1), while adjusting for relevant covariates. In addition, Cohen’s *d* effect sizes were computed to interpret the magnitude of all associations. Hemphill's (2003) interpretive framework for effect sizes, derived from an empirical assessment of the magnitude of the average effect sizes produced in psychological studies, was used to interpret effect size magnitudes. The correlational effect size guidelines provided by Hemphill (2003) were converted into Cohen's *d* effect sizes (Ruscio, 2008). Cohen's *d* effect sizes below 0.4 were considered small in magnitude, effect sizes between 0.4 and 0.6 were considered moderate, and effect sizes above 0.6 were considered large.

To preserve power and minimize missing data, participants were only compared during the first 8 weeks of treatment/waitlist. This is because data was only collected for half of the participants for 8 weeks (i.e., participants were either assigned to 8 or 16 weeks, and thus observations corresponding to weeks 9-16 would be missing for half of the sample due to the study design). A separate analysis wherein only participants assigned to a 16-week treatment intervention was conducted to corroborate the findings of the present analysis (see Table S1, Supplementary Materials). Moreover, since data was stratified by treatment group assignment and the experiment was conducted over several weeks, a heteroscedasticity and autocorrelation consistent (HAC) covariance matrix estimation was used to obtain a robust estimation of the linear models' standard errors (Cribari-Neto & da Silva, 2011).

Additionally, due to a large amount of missing observations in the dataset (i.e., 53% of observations for depression and anxiety), the data was also modeled using a Full Information Maximum Likelihood (FIML) estimation (cf. Hesser, 2015; Hoffart et al., 2022). FIML estimation allows for parameter estimates despite missing data by estimating patterns of missingness (Baraldi & Enders, 2010). This additional analysis was conducted to assess the convergence between FIML estimation and HAC covariance matrix estimation (i.e., compare the results obtained from the two methods). Isomorphic parameter estimates from both methods (i.e., in terms of signs and significance) will be taken as indicators of a stable and generalizable parameter estimation. In an effort to approach a model that might suggest potential causal effects of the war outbreak on anxiety and depressive symptom severity, all variables considered relevant were included in the analysis (Rohrer, 2018). A directed acyclic graph of the hypothesized causal associations and interdependencies in the assumed data-generating process was constructed using DAGitty to guide the choice of variables to adjust and not to adjust for in the present analysis (see Figure S2, Supplementary Materials; Textor et al., 2016).

## Results

### Sample Characteristics

Descriptive statistics for the sample demographics are summarized in Table 1.

**Table 1:** Demographical Descriptive Statistics

Participant Characteristics	Waitlist^a^, *n* = 560	Psychodynamic Affect Phobia Therapy^b^, *n* = 348	Cognitive Behavior Therapy^c^, *n* = 314	Total, *n* = 1,222
**Age**	43 (12)	43 (12)	44 (13)	42 (12)
Education				
Elementary School	14 (2.5%)	13 (3.7%)	7 (2.2%)	34 (2.8%)
High School	128 (23%)	89 (26%)	84 (27%)	301 (25%)
College-level education (< 3 years)	155 (28%)	96 (28%)	80 (25%)	331 (27%)
College-level education (> 3 years)	263 (47%)	150 (43%)	143 (46%)	556 (45%)
Sex				
Female	483 (86%)	302 (87%)	270 (86%)	1,055 (86%)
Male	73 (13%)	45 (13%)	43 (14%)	161 (13%)
Other	4 (0.7%)	1 (0.3%)	1 (0.3%)	6 (0.5%)
Self-rated socioeconomic status				
Much worse than others	23 (4.1%)	20 (5.7%)	12 (3.8%)	55 (4.5%)
Worse than others	132 (24%)	88 (25%)	60 (19%)	280 (23%)
About the same as others	234 (42%)	149 (43%)	145 (46%)	528 (43%)
Better than others	152 (27%)	86 (25%)	80 (25%)	318 (26%)
Much better than others	19 (3.4%)	5 (1.4%)	17 (5.4%)	41 (3.4%)
Children under 18 in the house				
No	347 (62%)	195 (56%)	191 (61%)	733 (60%)
Yes	206 (37%)	144 (41%)	116 (37%)	466 (38%)
Complicated/Sometimes	7 (1.2%)	9 (2.6%)	7 (2.2%)	23 (1.9%)
**Prior medication for anxiety/depression**	150 (27%)	85 (24%)	77 (25%)	312 (26%)
Current occupation				
Working	394 (70%)	226 (65%)	220 (70%)	840 (69%)
Studying	71 (13%)	48 (14%)	45 (14%)	164 (13%)
Seeking work	32 (5.7%)	19 (5.5%)	11 (3.5%)	62 (5.1%)
Retired	26 (4.6%)	20 (5.7%)	15 (4.8%)	61 (5.0%)
Parental leave	5 (0.9%)	8 (2.3%)	2 (0.6%)	15 (1.2%)
Sick leave	32 (5.7%)	27 (7.8%)	21 (6.7%)	80 (6.5%)

^a^Aggregated from four groups: Waitlist for 8 weeks, with discussion forum access (*n* = 126), Waitlist for 8 weeks, no discussion forum access (*n* = 122), Waitlist for 16 weeks, with discussion forum access (*n* = 154), Waitlist for 16 weeks, no discussion forum access (*n* = 158).

^b^Aggregated from four groups: Affect Phobia for 8 weeks, with discussion forum access (*n* = 59), Affect Phobia for 8 weeks, no discussion forum access (*n* = 61), Affect Phobia for 16 weeks, with discussion forum access (*n* = 111), Affect Phobia for 16 weeks, no discussion forum access (*n* = 117).

^c^Aggregated from four groups: Unified Protocol for 8 weeks, with discussion forum access (*n* = 46), Unified Protocol for 8 weeks, no discussion forum access (*n* = 58), Unified Protocol for 16 weeks, with discussion forum access (*n* = 100), Unified Protocol for 8 weeks, no discussion forum access (*n* = 110).

During screening, PHQ-9 scores ranged from 1 to 19 (*M* = 11.76, *SD* = 4.16), and GAD-7 scores ranged from 0 to 21 (*M* = 9.74, *SD* = 4.16). In the four weeks leading up to the war outbreak, PHQ-9 scores ranged from 0 to 27 (*M* = 8.84, *SD* = 5.14), and GAD-7 scores ranged from 0 to 21 (*M* = 7.79, *SD* = 4.80). In the four weeks following the war outbreak, PHQ-9 scores ranged from 0 to 27 (*M* = 8.26, *SD* = 5.36), and GAD-7 scores ranged from 0 to 21 (*M* = 7.83, *SD* = 5.14).

#### Missing Data

For the eight instances when data was provided, a Fisher's exact test comparing the propensity for data being differentially missing between the first four and latter four instances revealed non-significant differences for both the PHQ-9 (*p* = .168) and GAD-7 (*p* = .204). Furthermore, no obvious trends of missingness were discernible as a function of age, gender, or SES.

### The Effects of the War Outbreak

#### Symptoms of Depression in Response to the Outbreak

**Figure 1:**
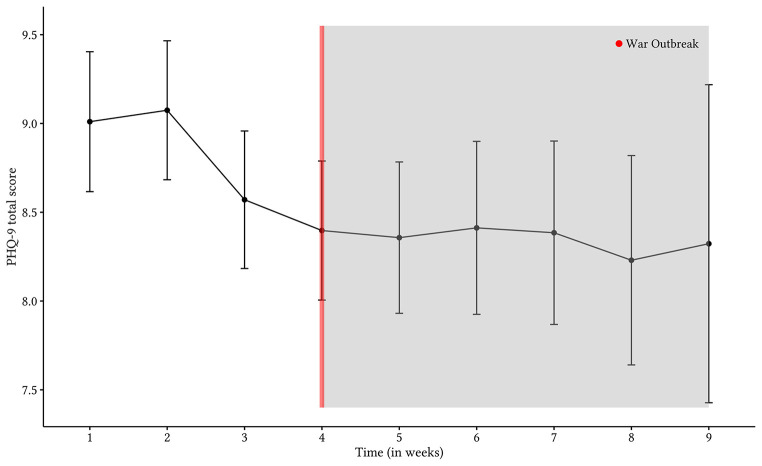
Graphical Depiction of Unadjusted Raw-Mean Scores and 95% Confidence Intervals for Depression Each Week, Over the Course of Treatment for All Treatment Groups

The outbreak of war did not significantly increase average levels of depression. Scores on the PHQ-9 slightly increased following the war outbreak, *t*(4566) = 0.39, *p* = .699, wherein comparing two individuals of the same socioeconomic status, treatment group, education level, age, and gender, while adjusting for the date on which data was provided, revealed a 0.09-point increase in average levels of depression, 95% CI [-0.38, 0.56]; effect size: *d* = 0.01, following the outbreak of war. The data was most compatible with values ranging from a 0.38-point decrease to a 0.56-point increase in scores on the PHQ-9. As such, the results do not indicate that the war outbreak significantly affected the severity of depression (see Figure 1).

#### Symptoms of Anxiety in Response to the Outbreak

**Figure 2:**
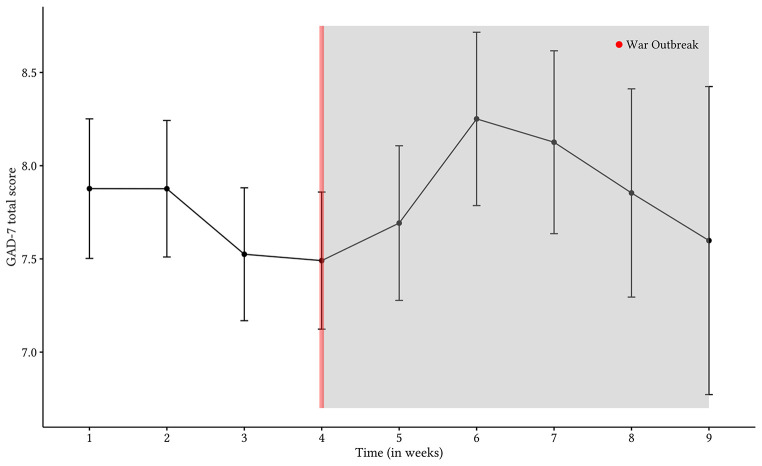
Graphical Depiction of Unadjusted Raw-Mean Scores and 95% Confidence Intervals for Anxiety Each Week, Over the Course of Treatment for All Treatment Groups

The war outbreak significantly increased average anxiety levels. Anxiety scores on the GAD-7 rose following the outbreak, *t*(4566) = 3.23, *p* = .001. Comparing two individuals with the same socioeconomic status, treatment group, education level, age, and gender, and adjusting for the data collection date, a 0.77-point increase in anxiety severity, 95% CI [0.30, 1.23]; effect size: *d* = 0.08, was observed after the war outbreak. The data was most compatible with values ranging from a 0.30-point to a 1.23-point increase in GAD-7 scores. A general decline in anxiety symptom severity was detected prior to the war outbreak which then increased abruptly in the wake of the war outbreak before rapidly declining to pre-war outbreak levels (see Figure 2). Consequently, the results suggest that the war outbreak exacerbated anxiety severity.

#### Socioeconomic Status as a Protective Factor

Socioeconomic status was inversely associated with anxiety severity over time, *t*(4566) = -3.61, *p* < .001, during this study. When comparing two individuals on the same date, within the same treatment group, of the same age, gender, and education level, while adjusting for the outbreak of war, a 1-point increase in self-rated socioeconomic status was associated with a 0.69-point average decrease in scores on the GAD-7, 95% CI [-1.06, -0.31]; effect size: *d* = 0.32. The data was most compatible with values ranging from a 1.06-point decrease to a 0.31-point decrease in scores on the GAD-7. Thus, anxiety symptom severity is, on average, lower for people with relatively higher socioeconomic status when controlling for the time course of treatment, war outbreak, gender, and treatment group, in turn, suggesting that socioeconomic status may be a potential protective factor for anxiety symptoms during a war outbreak (cf. entropy increase). Adding an interaction term between the war outbreak dummy variable and socioeconomic status did not increase the model fit nor alter the coefficient estimates.

Socioeconomic status was also inversely associated with depression severity over time, *t*(4566) = -5.28, *p* < .001, during this study. When comparing two individuals on the same date, within the same treatment group, of the same age, gender, and education level, while adjusting for the outbreak of war, a 1-point increase in self-rated socioeconomic status was associated with a 1.09-point average decrease in scores on the PHQ-9, 95% CI [-1.49, -0.68]; effect size: *d* = 0.48. The data was most compatible with values ranging from a 1.49-point decrease to a 0.68-point decrease in scores on the PHQ-9. Thus, depressive symptom severity is, on average, lower for people with relatively higher socioeconomic status when controlling for the time course of treatment, war outbreak, gender, and treatment group, in turn, suggesting that socioeconomic status may be a potential protective factor for depressive symptoms during a war outbreak (cf. entropy increase). Adding an interaction term between the war outbreak dummy variable and socioeconomic status did not increase the model fit nor alter the coefficient estimates.

### Additional Analyses

#### Full Information Maximum Likelihood (FIML) Estimation

To further support the previously reported results, linear models for the PHQ-9 and GAD-7 were analyzed using FIML estimations (see Table S2, Supplementary Materials). This analysis produced parameter estimates that were consistent with HAC covariance matrix estimation results reported earlier (i.e., equivalent parameter estimates and *p*-values). Moreover, an additional analysis that adjusted for all background variables at our disposal also produced parameter estimates that were consistent with both the HAC covariance matrix estimation and FIML results. Taken together, the parameter estimates seem stable in the current analysis, and patterns of missing data do not appear to significantly impact the results.

#### Treatment Group and Treatment Efficacy Analyses

Analyses of the differential effects of the war outbreak and overall treatment efficacy were conducted (see Supplementary Materials).

## Discussion

The present study aimed to elucidate the effects of the outbreak of war in Ukraine following the Russian invasion on February 24th on measures of anxiety and depressive symptom severity. To our knowledge, this is the first study on the indirect effects of the war in Ukraine on emotional disorders in a clinical population, thereby providing up-to-date information about the reaction of treatment-seeking individuals to uncertainty-inducing events. The results indicate that anxiety symptoms significantly increased in response to the war outbreak, as predicted, although this effect was small in magnitude (cf. Hemphill, 2003). Anxiety symptom severity generally declined before the outbreak of war, spiked following the war outbreak, before rapidly declining to pre-war outbreak levels. However, contrary to our hypothesis, the war outbreak had a negligible effect on depressive symptoms. Depressive symptoms gradually declined throughout the duration of the study and did not spike in response to the war outbreak. Finally, socioeconomic status had a moderate effect on decreased anxiety symptoms and decreased depressive symptoms over the course of treatment, irrespective of the war outbreak. These findings thus provide support for the notion that socioeconomic status serves as a protective factor against psychopathology in times of heightened uncertainty.

The finding that anxiety symptom severity increased in response to the war outbreak, but depressive symptom severity did not, may relate to how anxiety and depression are differentially associated with intolerance of uncertainty. As noted in the introduction, intolerance of uncertainty, which may underpin many psychopathological impairments to daily functioning, has been suggested to be more pronounced in anxiety disorders than depression (Jensen et al., 2016). However, meta-analytic findings suggest that intolerance of uncertainty lacks etiological specificity to differentiate anxiety and depression (Gentes & Ruscio, 2011). Nonetheless, the semantic link between anxiety and intolerance of uncertainty is reflected in the American Psychiatric Association's (2022, p. 215) definition of anxiety as the "anticipation of [a] future threat," which coincides with the definition of intolerance of uncertainty (i.e., responding to uncertainty-inducing events with discomfort and anxiety which, in turn, further increases negative affectivity, cf. psychological entropy; Hirsh et al., 2012; Jensen et al., 2016). Furthermore, even though the effect of the war outbreak on anxiety symptoms is small in magnitude by most statistical standards, it is important to consider the clinical implications of uncertainty-inducing events on anxiety symptoms within a treatment-seeking population and place the effect in a broader context. For instance, the magnitude of the effect between increased anxiety symptoms in response to the war outbreak is slightly larger than the association between aspirin consumption and heart attack prevention (Rosenthal, 1991, p. 136; see also Hemphill, 2003). Moreover, this effect size mirrors typical effect sizes that research on the effect of disasters on mental health disorders produces, where pooled effect estimates range from 0.05 and 0.20 (Keya et al., 2023).

The present study has limitations. In line with previous studies (e.g., Guerra & Eboreime, 2021; Skwirczyńska et al., 2022), we found socioeconomic status to buffer against psychopathological development following the abrupt increase in external uncertainty due to the war outbreak. However, the interpretation of this effect may be limited by using self-reported socioeconomic status, where participants self-rated their socioeconomic status in relation to others. Another limitation is our lack of control for media exposure. Previous studies indicate frequency of media exposure to covary with anxiety and depression symptom severity (Gao et al., 2020; Riad et al., 2022). As such, without control for participant exposure to media coverage of the war, effects of the war outbreak on anxiety and depression symptom severity may have been attenuated (or even augmented).

Furthermore, this study is limited by a lack of qualitative interviews to provide insight into participant's experiences and perceptions of the war outbreak and its effects on their mental health. Future studies could ameliorate this limitation by incorporating an ecological momentary assessment protocol (e.g., Verhagen et al., 2022), wherein data on exposure to war-related media and self-reported affectedness of the war outbreak is collected with high frequency concomitantly with indices of anxiety and depression. No clinical interviews were conducted to accurately detect whether participants qualified for a diagnosis of an anxiety or depressive disorder. However, only treatment-seeking participants with scores indicative of an emotional disorder were included in the study, and the PHQ-9 and the GAD-7 routinely emerge as good indicators of depressive and anxiety disorders (Byrd-Bredbenner et al., 2021; Johnson et al., 2019; Martin-Key et al., 2022). Additionally, this study is limited by design; temporal precedence was established but true causality cannot be inferred from the present analysis.

Finally, the large number of missing observations in the measures of anxiety and depression severity somewhat limits the statistical analyses. The possibility that participants selectively neglected to provide data when they suffered most severely from depression and/or anxiety cannot be eliminated. However, there were no discernible trends in the missingness of data. Moreover, modelling the data with state-of-the-art statistical procedures for handling missing data (i.e., FIML and robust HAC versions of the general linear model) did not influence the statistical conclusion of the results as it yielded isomorphic parameter estimations.

The present study has numerous strengths. Firstly, this study is the first analysis of the impact of the war in Ukraine in a clinical sample, and thus provides up-to-date information about the reaction of treatment-seeking individuals to abrupt uncertainty. Additionally, although greater average variability in indicators of depression and anxiety is to be expected in clinical samples (Hirsh et al., 2012; Sauer-Zavala & Barlow, 2021), a clear upward spike in average levels of depression and anxiety severity in response to the war outbreak was discernible. Secondly, measures of anxiety and depression severity were obtained weekly throughout the treatment intervention, allowing for a representative estimation of the psychopathological response to the war outbreak. Thirdly, given that psychopathological development surges in response to abrupt uncertainty-inducing events (cf. entropy increase; Guerra & Eboreime, 2021; Hirsh et al., 2012; Lim et al., 2022; Osokina et al., 2023; Riad et al., 2022), our study may have buffered psychopathological development among Swedish treatment-seeking individuals. Other strengths include the exclusive inclusion of treatment-seeking individuals and an adequately large sample size.

The present study may have implications for how abrupt uncertainty-inducing events can be mitigated at a population level. Briefly, our results suggest that anxiety symptom severity rises in conjunction with increased environmental uncertainty (cf. entropy increase); a particularly interesting finding considering the geographical distance between Sweden and Ukraine, which exceeds 1500 km. The study underscores the need for heightened vigilance and support for individuals predisposed to psychopathology when confronted with sudden, uncertainty-inducing events, irrespective of their physical proximity. However, it is important to approach these findings with caution. The study did not directly measure participants' perceptions of the war outbreak or ascertain which specific aspects of the conflict were most impactful to them. Given this limitation, the direct influence of the war outbreak on the observed increase in anxiety symptoms remains speculative. Nevertheless, providing readily accessible health care services, such as government-funded internet-based psychotherapy, in the aftermath of such events could be beneficial. This approach may help alleviate societal impacts and reduce the overall burden of such events, particularly for individuals with below-average socioeconomic status who might encounter additional challenges in the wake of uncertainty-inducing events.

Finally, this study holds implications for clinicians in practice. It suggests that when psychotherapy is provided during crises, a sudden increase in anxiety symptoms can, in general, be expected in response to heightened environmental uncertainty (cf. entropy increase). However, statistically controlling for this crisis-related increase reveals that overall severity of anxiety symptoms continues to decrease throughout the course of treatment. As such, an increase in anxiety symptoms during crises situations should not automatically be interpreted as an indicator of unsuccessful treatment. Instead, it should be recognized as a potential confounding factor in estimating treatment efficacy. Furthermore, this effect differed between treatment group assignments (see Figures S3 and S4, Supplementary Materials).

### Conclusion

The present study highlights the impact of the Ukrainian war outbreak on emotional disorders, particularly anxiety symptoms, in a clinical population. Anxiety symptom severity seems to be sensitive to the conflict's influence, experiencing an increase of up to 1.22 points following the war outbreak. Moreover, socioeconomic status may serve as a protective factor against the development of psychopathological disorders in the wake of uncertainty-inducing events. Lastly, this study reinforces previous findings demonstrating the effectiveness of internet-based psychotherapeutic interventions in alleviating emotional disorder symptoms.

## Supplementary Materials

The Supplementary Materials include the following items (see Hlynsson et al., 2024):

- A flow chart illustrating the study design.
- An analysis featuring participants exclusively assigned to a 16-week treatment intervention to corroborate the findings reported in this paper.
- A Directed Acyclic Graph illustrating the hypothesized causal model and associations between variables.
- Full Information Maximum Likelihood (FIML) estimates contrasted with the heteroscedasticity and autocorrelation consistent (HAC) covariance matrix estimation.
- An analysis of the effect of treatment group assignment during the study period.
- An assessment of overall treatment efficacy during the study period.

HlynssonJ. I.
GustafssonO.
CarlbringP.
 (2024). Supplementary materials to "Uncertainty breeds anxiety and depression: The impact of the Russian invasion in Ukraine on a Swedish clinical population receiving internet-based psychotherapy"
[Additional information]. PsychOpen. 10.23668/psycharchives.14140
PMC1130391139119223

## Data Availability

The data that support the findings of this study are available from the corresponding author, JIH, upon reasonable request.

## Preregistration

This study was not preregistered.
